# Country's value priorities in health crisis: How dominant societal motivations shape COVID-19 severity

**DOI:** 10.1016/j.ssmph.2023.101493

**Published:** 2023-08-19

**Authors:** Mac Zewei Ma, Shengquan Ye

**Affiliations:** a Department of Applied Social Sciences, The Hong Kong Polytechnic University, PR China; b Department of Social and Behavioural Sciences, City University of Hong Kong, PR China

**Keywords:** *COVID-19*, *Circular model of human values*, *Self-enhancement values*, *European social survey*, *World values survey*, *Archival indicators*

## Abstract

This paper presents two comprehensive studies examining how Schwartz's human values dimensions at the country level predict COVID-19 pandemic severity. Study 1 aggregated survey data across 89 countries from the European Social Survey and World Values Survey to assess societal-level conservation versus openness to change (CON-OTC) and self-enhancement versus self-transcendence (SE-ST) value-continuums. Study 2 developed an innovative archival measurement approach using 10 indicators to estimate these value dimensions for over 180 countries. Both studies employed multilevel modeling to test the relationships between country-level values and COVID-19 severity, measured through epidemiological indicators of transmission speed, case fatality rate, infection prevalence and mortality burden. Results revealed that the CON-OTC and SE-ST value-continuums showed consistent, significant negative associations with transmission speed and infection prevalence before adjusting for modernization, latitude, historical pathogen prevalence and government stringency across both studies. However, after accounting for these socioecological and policy covariates, the CON-OTC value-continuum positively predicted case fatality rate in both studies, implying conservation values could increase COVID-19 lethality. In contrast, across both studies, the SE-ST value-continuum negatively predicted case fatality rate after adjusting for the covariates, suggesting countries prioritizing self-enhancement values exhibited relatively lower pandemic severity and lethality when accounting for developmental, ecological, and policy factors. Accordingly, the studies advance theoretical understanding of how country's value priorities shape COVID-19 impact. Methodologically, these studies contribute through multilevel techniques that account for spatial dependencies, as well as an innovative ecological measurement. Overall, this research demonstrates the value of applying Schwartz's framework at a societal level to predict global health crises and pandemics.

## Highlights

• Examined the link between country-level Schwartz's human values and COVID-19 severity in two large-scale studies.
• Used archival indicators for innovative assessment of Schwartz's values at the country level.
• Employed multilevel modeling for spatial dependencies in regional clusters.
• Identified Schwartz's value-continuums impacting COVID-19 severity.
• Highlighted the efficacy of Schwartz's values in predicting national pandemic outcomes.

## Funding

This work was supported by the Start-up Fund for Research Assistant Professors under the Strategic Hiring Scheme of The 10.13039/501100004377Hong Kong Polytechnic University [grant number P0046376] awarded to Mac Zewei Ma.

## 1 Introduction

The parasite-stress theory proposes that cultural values evolve as adaptive responses to disease threats, with collectivism values potentially functioning as disease-avoidance mechanisms (Fincher et al., 2008; Fischer & Poortinga, 2012; Thornhill et al., 2010). Thus, examining cultural values through this theoretical lens is essential for understanding the COVID-19 pandemic (Ma, 2021, 2022b; Ma & Ye, 2021). Studies have shown collectivism values positively predict COVID-19 responses at individual and societal levels (Biddlestone et al., 2020; Canatay et al., 2021; Cho et al., 2022; Dang & Xiao, 2022; Germani et al., 2020; Gokmen et al., 2021; Huang et al., 2020; Lu et al., 2021; Maaravi et al., 2021; Rajkumar, 2021; Webster et al., 2021). However, the broad, multifaceted nature of collectivism and individualism (Talhelm & English, 2020) limits understanding of the specific relevance of cultural values to COVID-19 pandemic.

Schwartz's human values model (1994), which has empirical support in cross-national and experimental studies (Schwartz et al., 2001, 2012; Schwartz & Rubel-Lifschitz, 2009), could address this limitation (Clay et al., 2012). As fundamental motivational goals, values play a significant role in guiding human behavior (Schwartz, 1994, 2012). Wolf et al. (2020) highlight Schwartz's values' importance amid the COVID-19 pandemic. Thus, investigating how country-level human values, measured through Schwartz's framework, predict COVID-19 outcomes aligns with the parasite-stress theory's propositions.

Schwartz's model organizes ten basic human values hierarchically based on motivational goals (Schwartz, 1994). Four higher-order value dimensions emerge from the compatibility between basic values: openness to change (OTC), conservation (CON), self-enhancement (SE), and self-transcendence (ST). OTC combines the values of self-direction, stimulation, and hedonism. CON comprises security, conformity, and tradition. SE represents power and achievement, while ST combines universalism and benevolence. Two key value-continuums underpin the circular structure: conservation versus openness to change (CON-OTC) and self-enhancement versus self-transcendence (SE-ST) (Boer & Fischer, 2013).

According to Schwartz (2012), CON and SE values motivate controlling anxiety-arousing threats and defending against real-world uncertainty (Schwartz et al., 2012). For instance, tradition and security uphold order, conformity avoids conflict, and power and achievement enable active threat control by acquiring resources and demonstrating competence. In contrast, OTC and ST synergistically promote anxiety-free motivational goals for self-expansion and growth (Schwartz et al., 2012). Their basic values, like self-direction, stimulation, universalism, and benevolence, all facilitate such objectives.

Within the COVID-19 context, certain Schwartz's values may act as specific disease-avoidance strategies, particularly conservation and self-enhancement. The adjacent conservation values of tradition and security motivate upholding social norms (Schwartz, 2012). Individuals may thus adhere to hygiene habits like pre-meal handwashing to prevent coronavirus transmission. Relatedly, the self-protective motivations of achievement and power drive safety maintenance through resource and relationship control (Schwartz, 2012), which could also reduce contagion (Clay et al., 2012). Notably, combining power values with collectivism yielded the construct of vertical collectivism (Triandis, 1996). Further evidence indicates vertical collectivism is positively associated with conservation and self-enhancement values (Clay et al., 2012; Soh & Leong, 2002)—an orientation linked to greater COVID-19 concern and control intentions (Biddlestone et al., 2020; Cho et al., 2022; Dang & Xiao, 2022; Germani et al., 2020; Huang et al., 2020; Lu et al., 2021). Vertical collectivism is proposed as an evolutionarily-developed disease-avoidance strategy (Clay et al., 2012), suggesting conservation and self-enhancement values may function as infectious disease defenses (Ackerman et al., 2018). Supporting this, positive correlations between pathogen prevalence and these values were found, although modernization requires consideration as a potential confound (Ma, 2020).

However, amid COVID-19, the self-growth, anxiety-free OTC may not effectively avoid disease. OTC values like hedonism and stimulation seek pleasant arousal (Schwartz, 2012) but may unintentionally increase coronavirus transmission risks. Moreover, the independent thought and action of high self-direction could reduce compliance with strict COVID-19 preventives like stay-at-home orders.

Moreover, the role of ST values in COVID-19 prevention and control remains uncertain. While research indicates ST values may mitigate COVID-19 threat (Ma, 2022a; Selçuk & Grassie, 2023; Wolf et al., 2020), other studies paint a more complex picture. For instance, Tabernero et al. (2020) found individuals with higher ST values reported less COVID-19 coping, implying ST may not consistently aid pandemic control. In contrast, the self-protective SE values, exemplified by the basic value of power, positively predicted engagement in coping behaviors and physical distancing (Tabernero et al., 2020). This aligns with SE's active threat control motivation (Schwartz, 2012; Schwartz et al., 2012). Therefore, the relationship between ST values and COVID-19 responses seems context-dependent.

Recent research reveals spatial disparities in COVID-19 cases and deaths across countries (Banik et al., 2020; Hilton & Keeling, 2020; Jankowiak et al., 2020; Kapitsinis, 2020). Proposed contributing factors include climate, ecology, GDP per capita, population density, and airport traffic (Gangemi et al., 2020; Jankowiak et al., 2020; Notari, 2021). Although Hofstede's (1980) cultural dimensions have been applied to understand country-level COVID-19 susceptibility (Gokmen et al., 2021), they have limitations from outdated data and measurement issues.

Schwartz's circular human values model offers an updated, validated framework shown to be consistent across cultures (Schwartz, 1994; Schwartz & Bardi, 2001). Since human values theoretically and empirically predict COVID-19 responses (Ma, 2022a; Selçuk & Grassie, 2023; Tabernero et al., 2020; Wolf et al., 2020), examining country-level values' predictive ability is important. Since Schwartz's values manifest across major cultures with substantial similarity in value structures across levels (Fischer & Poortinga, 2012), applying Schwartz's model at the country level could offer valuable insights into the relationship between group-level values and COVID-19 severity.

To address the research gap, two comprehensive studies are conducted. Study 1 utilizes the self-reported data from European Social Survey and World Values Survey to capture human values across countries. Study 2 employs 10 archival indicators to assess Schwartz's values at the country level, offering an alternative measurement approach to enhance robustness of the findings (Bardi et al., 2008). This research utilizes various epidemiological indicators and controls for potential confounds to comprehensively understand the association between country-level values and COVID-19 severity. Both studies employ multilevel analysis techniques to account for geographical dependencies.

Drawing on Schwartz's motivational goals and existing theoretical and empirical evidence linking values to COVID-19 pandemic, hypotheses are developed to elucidate how country-level values may influence pandemic severity.

H1 *The value-continuum of CON–OTC is hypothesized to have a negative association with COVID-19 severity*.

Countries prioritizing conservation values like security, conformity, and tradition are expected to exhibit lower COVID-19 severity. These values motivate self-protection, which might contribute to reduced transmission.

H2 *The value-continuum of SE–ST is hypothesized to have a negative association with COVID-19 severity*.

Countries emphasizing self-enhancement values such as power and achievement are expected to demonstrate lower COVID-19 severity. These values drive active threat control and resource acquisition, potentially increasing preventive measure adherence and proactive pandemic responses.

This research addresses the significant gap in understanding country's value priorities in health crisis. Through two comprehensive studies utilizing self-reported data (Study 1) and archival indicators (Study 2), it aims to elucidate how country-level values contribute to cross-national variability in COVID-19 outcomes. Focusing on the CON-OTC and SE-ST value-continuums could inform policymakers and professionals in developing targeted strategies to mitigate pandemic impacts. Moreover, advancing knowledge on values' roles in shaping COVID-19 responses could also provide insights to aid global efforts combating the virus.

## 2 Study 1

### 2.1 Method

#### 2.1.1 Countries and timeframe

**Fig. 1:**
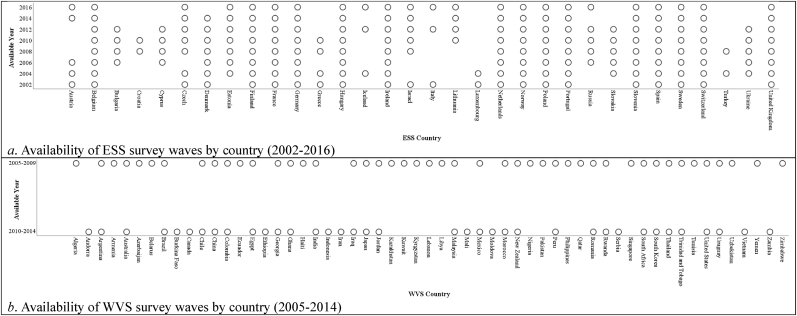
Availability of ESS and WVS survey waves by country.

This study utilized data from the European Social Survey (ESS) from 2002 to 2016, providing 374,730 responses across 32 countries, and the World Values Survey (WVS) from 2005 to 2014, contributing 129,182 responses across 57 countries. In total, 89 countries were included. Fig. 1 displays the availability of ESS and WVS survey waves by country. COVID-19 epidemiological data spanning January 2020 to December 2022 were obtained from Our World in Data. All research data for this study can be accessed via this Open Science Framework (OSF) repository: https://osf.io/jgdfm/?view_only=c65131f8890c4419ba56a788e99f1084.

#### 2.1.2 Measures

##### 2.1.2.1 Country-level values

Country-level values were obtained by aggregating individual responses from the ESS and WVS, with weighting scores provided by the respective research (Ma, 2020). The ESS used a 21-item short version of the Portrait Values Questionnaire (PVQ) (Schwartz et al., 2001) in all survey rounds, while the WVS used a 10-item PVQ for Round 5 and an 11-item PVQ for Round 6 (Inglehart et al., 2020). Respondents indicated their agreement or disagreement on a 6-point Likert scale regarding statements describing personal values. Scores were reverse-coded, with higher scores indicating greater importance placed on those values. The CON, OTC, SE, and ST value dimensions were calculated from both datasets. Internal consistency was assessed using Cronbach's alpha, which was 0.73 for CON, 0.77 for OTC, 0.73 for SE, and 0.73 for ST in ESS, and 0.62 for CON, 0.52 for OTC, 0.52 for SE, and 0.73 for ST in WVS.

**Fig. 2:**
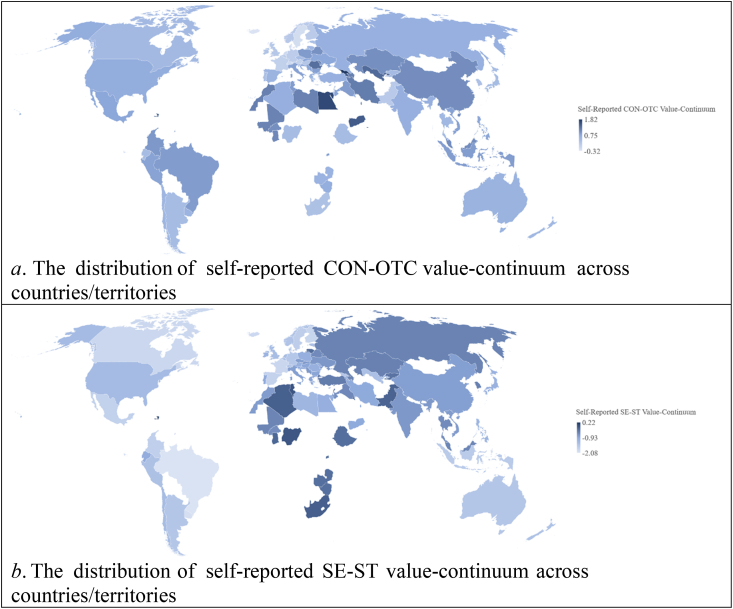
Distribution of self-reported value-continuums across 89 countries/territories in Study 1.

To determine the relative importance of values for respondents, individual-level scores were ipsatized by subtracting the grand mean from raw values. Positive ipsatized scores indicate higher importance placed on specific values. These scores were aggregated across countries to create country-level value dimensions for CON, OTC, SE, and ST. Country-level analysis revealed strong negative relationships between CON and OTC (*r* = −0.78, *p* < .001) and between SE and ST (*r* = −0.79, *p* < .001), consistent with Schwartz's higher-order conflicting values dimensions (Schwartz, 2012). To simplify the structure, value-continuums were calculated: CON-OTC representing conservation versus openness to change (subtracting OTC from CON) and SE-ST representing self-enhancement versus self-transcendence (subtracting ST from SE) at the country level. Fig. 2 displays the distribution of these value-continuums across 89 countries/territories.

##### 2.1.2.2 COVID-19 severity indicators

Rather than creating a composite index of COVID-19 severity, this study examined the relationships between values and various indicators of COVID-19 severity. This approach could provide a more detailed and specific understanding of the roles of values during the pandemic.

The reproductive ratio of the SARS-CoV-2 virus was used to measure the speed of COVID-19 transmission. It represents the average number of secondary cases expected from an average primary case in a fully susceptible population (Held et al., 2019). A daily average reproductive ratio was calculated, with higher values indicating a faster spread of the virus.

The case fatality ratio was employed to assess the lethality of COVID-19. This ratio captures the “killing power” of infectious diseases (Hiremath, 2011) and was calculated as (daily COVID-19 deaths per million/daily COVID-19 cases per million) × 100. A higher daily average case fatality ratio signifies a greater lethality of the novel coronavirus.

In addition, the prevalence rate and mortality rate of COVID-19 were measured using COVID-19 cases (per million) and deaths (per million), respectively. These daily average values were computed for each country/territory.

##### 2.1.2.3 Covariates

First, the government stringency index (GSI) was calculated as the daily average using data from the University of Oxford's COVID-19 Government Response Tracker, measuring government policy stringency (Frey et al., 2020; Kapoor et al., 2021; Sorci et al., 2020; Wang, 2021). Second, latitude was included to control for climatic factors like temperature (Notari, 2021). Third, the historical infectious disease prevalence index (Murray & Schaller, 2010) controlled for factors related to novel disease outbreaks (Jankowiak et al., 2020; Thornhill et al., 2010). Finally, modernization indicators associated with COVID-19 prevalence and mortality were considered (Gangemi et al., 2020; Jankowiak et al., 2020; Roy & Ghosh, 2020). Following the “law of briefness”, Ma's (2020) modernization index was utilized, capturing modernization's multifaceted nature (Stockemer & Sundström, 2016).

### 2.2 Statistical approach

A bivariate correlation analysis was initially conducted to examine the simple correlations between values and COVID-19 severity indicators, assessing their basic relationships.

Considering the issue of spatial autocorrelation, where variables tend to cluster in nearby locations (Dormann et al., 2007; Gu et al., 2020; Kerkman et al., 2017; Legendre, 1993), multilevel analysis was employed. Geopolitical regions defined by the United Nations (https://unstats.un.org/unsd/methodology/m49/) were used for nesting countries/territories within corresponding regions (Kusano & Kemmelmeier, 2020; Ma, 2022b) to account for spatial clustering.

The suitability of multilevel analysis was assessed using the Likelihood-ratio test (LRT) and Intraclass Correlation Coefficient (ICC). An unconditional random-intercept multilevel model was initially calculated to determine the proportion of variance in COVID-19 severity attributable to geopolitical regions.

Multilevel models were computed to examine the unique effects of human values on COVID-19 severity. The first model considered only the effects of values, while the second incorporated all covariates to assess the unique predictive power of values related to COVID-19 severity after accounting for other factors.

The random-intercept multilevel model was represented by the following equations:

Level 1 (Country-level):

Y_*ij*_ = B_0*j*_ + B_1_ × Latitude_*ij*_ + B_2_ × Historical parasite-stress index_*ij*_ + B_3_ × Modernization index_*ij*_ + B_4_ × Stringency index_*ij*_ + B_5_ × CON-OTC value-continuum_*ij*_ + B_6_ × SE-ST value-continuum_*ij*_ + e_*ij*_

Level 2 (Geopolitical-region-level):

B_0*j*_ = γ_00_ + u_0*j*_

Where:

Y_*ij*_ represents the COVID-19 severity for country/territory *i* in geopolitical region *j*. B_0*j*_ represents the random intercept for geopolitical region *j*, indicating the baseline COVID-19 severity for that geopolitical region. B_1_ to B_6_ are the fixed-effect coefficients for the country-level predictors. γ_00_ represents the overall intercept for the geopolitical-region-level model. The e_*ij*_ is country-level residual term. The u_0*j*_ is geopolitical-region-level residual term. The model allows for the estimation of geopolitical-region-specific random intercepts (B_0*j*_) and the variation in those intercepts (u_0*j*_) while accounting for the effects of country-level predictors (B_1_ to B_6_) and the residual error (e_*ij*_).

### 2.3 Results and discussion

**Fig. 3:**
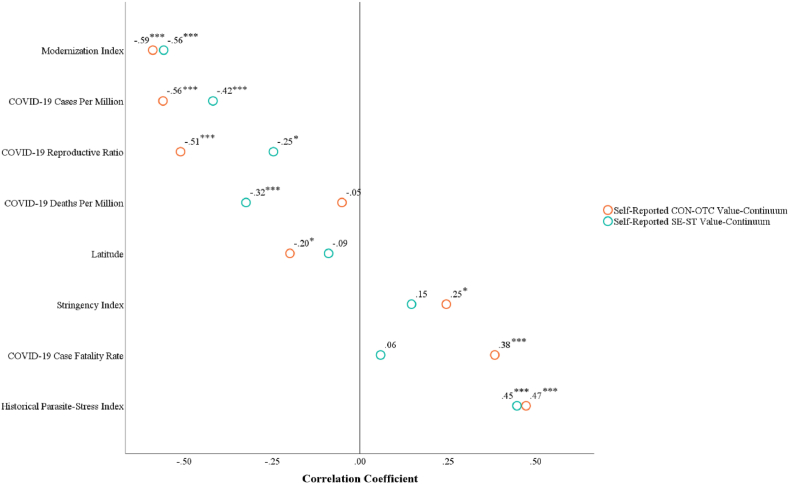
The correlations between country-level values and all variables in Study 1. Note. *p_one-tailed_ < 0.05, ***p_one-tailed_ < 0.001.

**Fig. 4:**
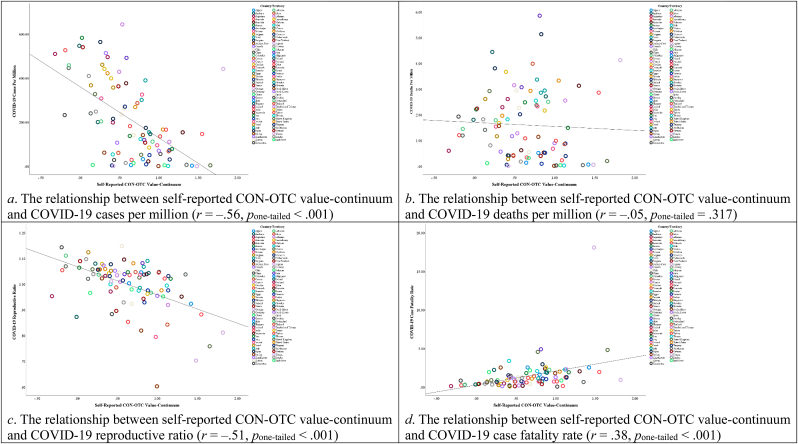
Scatter plots displaying the relationships between self-reported CON-OTC value-continuum and COVID-19 severity indicators in Study 1.

**Fig. 5:**
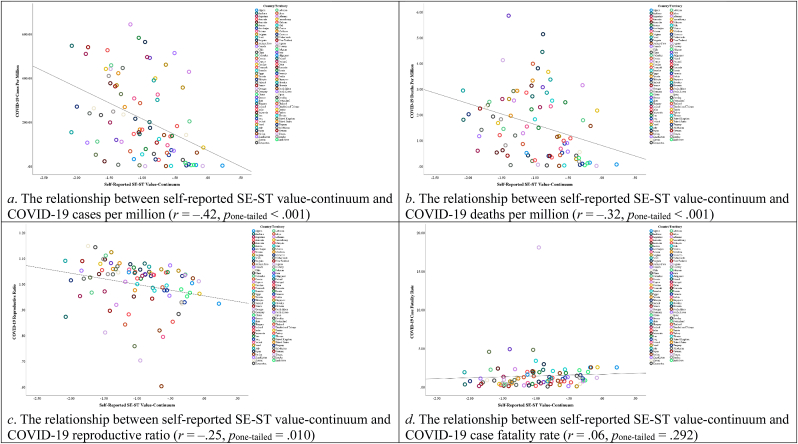
Scatter plots displaying the relationships between self-reported SE-ST value-continuum and COVID-19 severity indicators in Study 1.

Fig. 3 displays the correlations between values and all variables, with detailed descriptive statistics and correlational relationships provided in Table S1 of the Supplementary File. In addition, Fig. 4 shows that the CON-OTC value-continuum had a significant negative relationship with COVID-19 cases per million and the reproductive ratio, but a significant positive relationship with the case fatality rate. Fig. 5 demonstrates that the SE-ST value-continuum had a significant negative association with COVID-19 cases per million, COVID-19 deaths per million, and the reproductive ratio.

Unconditional multilevel models were used, with geopolitical regions serving as a random intercept. Geopolitical regions explained a significant proportion of the variance in COVID-19 cases per million (ICC = 54%, LRT = 36, *p* < .001), COVID-19 deaths per million (ICC = 47.6%, LRT = 32.1, *p* < .001), COVID-19 reproductive ratio (ICC = 24.4%, LRT = 10.3, *p* = .001), justifying the use of multilevel analyses (Hox, 2010; Lorah, 2018). Although geopolitical regions did not account for significant variance in COVID-19 case fatality rate (ICC = 0.43%, LRT = 0.01, *p* = .937), multilevel analysis was still conducted for this outcome variable to maintain consistency in the statistical reporting across the four COVID-19 severity indicators.

Results in Table S2 show that before controlling for covariates, the CON-OTC value-continuum significantly and negatively predicted COVID-19 cases per million (B = −173.64, *SE* = 36.68, *df* = 86, *t* = −4.73, *p*_one-tailed_ < 0.001), while the SE-ST value-continuum also significantly and negatively predicted COVID-19 cases per million (B = −86, *SE* = 29.60, *df* = 86, *t* = −2.91, *p*_one-tailed_ < 0.001). However, after controlling for covariates, neither value-continuum significantly predicted COVID-19 cases per million (CON-OTC: B = −44.36, *SE* = 33.31, *df* = 81, *t* = −1.33, *p*_one-tailed_ = 0.093; SE-ST: B = 7.78, *SE* = 25.83, *df* = 81, *t* = 0.30, *p*_one-tailed_ = 0.382).

The CON-OTC value-continuum did not have a significant effect on COVID-19 deaths per million before controlling for covariates (B = 0.23, *SE* = 0.31, *df* = 86, *t* = 0.76, *p*_one-tailed_ = 0.225), but became a significant predictor after controlling for covariates (B = 0.67, *SE* = 0.34, *df* = 81, *t* = 1.97, *p*_one-tailed_ = 0.026). The SE-ST value-continuum non-significantly predicted COVID-19 deaths per million before controlling for covariates (B = −0.35, *SE* = 0.25, *df* = 86, *t* = −1.42, *p*_one-tailed_ = 0.080), and it remained a non-significant predictor after accounting for covariates (B = 0.03, *SE* = 0.26, *df* = 81, *t* = 0.10, *p*_one-tailed_ = 0.461).

The CON-OTC value-continuum significantly and negatively predicted the COVID-19 reproductive ratio, both before controlling for covariates (B = −0.10, *SE* = 0.02, *df* = 86, *t* = −5.16, *p*_one-tailed_ < 0.001) and after controlling for covariates (B = −0.07, *SE* = 0.02, *df* = 81, *t* = −3.35, *p*_one-tailed_ < 0.001). However, the SE-ST value-continuum did not significantly predict the COVID-19 reproductive ratio, neither before controlling for covariates (B = −0.02, *SE* = 0.02, *df* = 86, *t* = −1.43, *p*_one-tailed_ = 0.077) nor after controlling for covariates (B = 0.002, *SE* = 0.02, *df* = 81, *t* = 0.13, *p*_one-tailed_ = 0.447).

The CON-OTC value-continuum significantly predicted the COVID-19 case fatality rate (B = 1.84, *SE* = 0.47, *df* = 86, *t* = 3.89, *p*_one-tailed_ < 0.001), which remained significant after controlling for covariates (B = 1.18, *SE* = 0.55, *df* = 81, *t* = 2.17, *p*_one-tailed_ = 0.016). Although the SE-ST value-continuum did not directly predict the COVID-19 case fatality rate (B = −0.13, *SE* = 0.39, *df* = 86, *t* = −0.34, *p*_one-tailed_ = 0.368), it became a significant predictor after controlling for covariates (B = −0.77, *SE* = 0.44, *df* = 81, *t* = −1.77, *p*_one-tailed_ = 0.040).

**Fig. 6:**
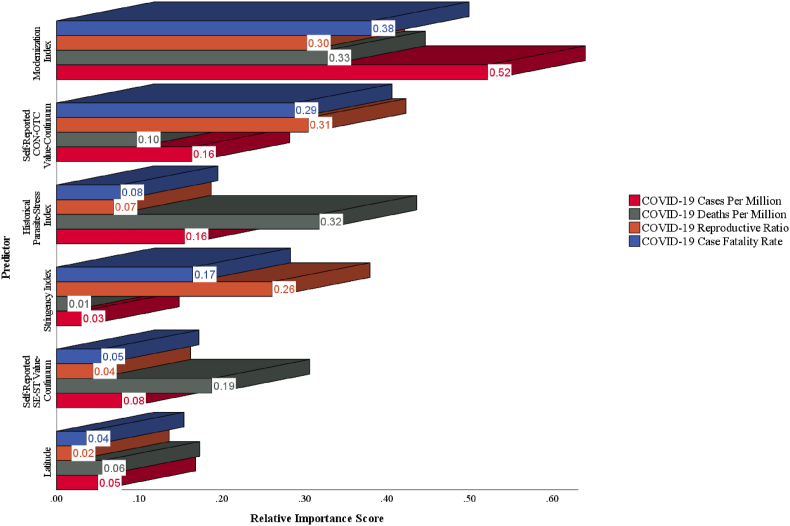
Relative importance of predictors for COVID-19 severity indicators in Study 1.

To determine the impact of human values and other covariates on COVID-19 severity indicators, the Lindeman, Merenda, and Gold method was used to calculate the relative importance score (Grömping, 2007). Fig. 6 shows that Ma's modernization index (2020) was the most important predictor of the overall severity of COVID-19 pandemic, highlighting the significant role of a country's modernization process in the occurrence and persistence of the pandemic. Additionally, the self-reported CON-OTC value-continuum was more important in predicting COVID-19 severity indicators than the self-reported SE-ST value-continuum. Specifically, the self-reported CON-OTC value-continuum had the highest relative importance score in predicting the COVID-19 reproductive ratio (0.31).

The study found after accounting for covariates, conservation-oriented countries had slower initial virus spread yet higher lethality, while self-enhancement-oriented countries saw lower lethality. However, limitations exist regarding the small sample size and indirect measurement of group values via surveys. To validate the results, a new ecological measure was developed using archival indicators to directly capture group-level values.

## 3 Study 2

Study 2 aimed to replicate Study 1 using a more ecological approach to assess country-level values. The research design and statistical methods mirrored Study 1, but group-level values were measured with archival indicators. Despite Schwartz (2011) advocating ecological measurement, few studies have employed this. Thus, this study introduced a novel measure utilizing theoretically-relevant archival indicators to assess Schwartz's values and explore how archive-based value-continuums predict COVID-19 severity across countries. This ecological approach could provide an alternative perspective to enhance robustness of the findings. Research data can be found via this OSF repository: https://osf.io/jgdfm/?view_only=c65131f8890c4419ba56a788e99f1084.

### 3.1 Method

#### 3.1.1 Countries and timeframe

This research utilized archival data to estimate the group-level CON-OTC and SE-ST value-continuums for 190 and 180 countries/territories, respectively. However, analysis of COVID-19 severity indicators included a varying number of countries due to unavailable epidemiological data for some regions.

#### 3.1.2 Measures

##### 3.1.2.1 Group-level values

**Fig. 7:**
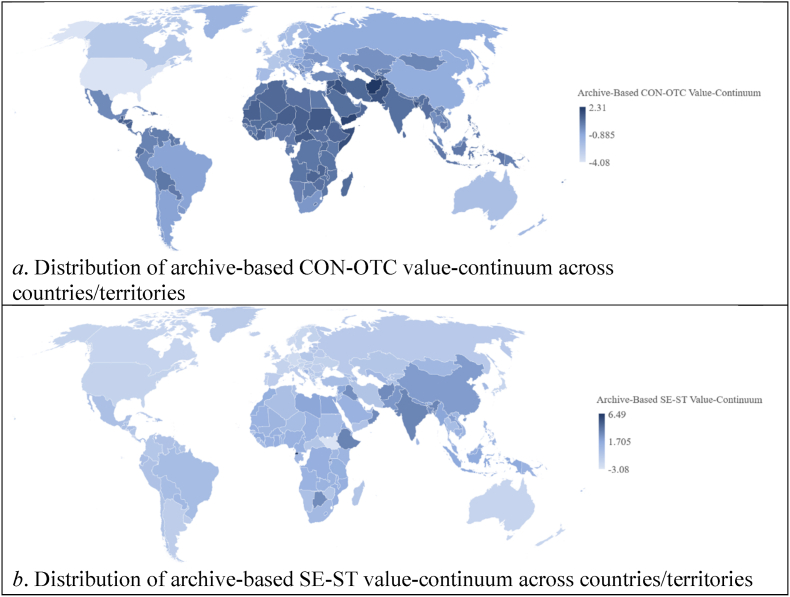
Distribution of archive-based value-continuums across the globe in Study 2.

Table 1 presents 10 archival indicators for group-level values, based on theoretical definitions. Table SS1 (see Supplementary File) details the rationale behind including the specific archival indicator(s). Ipsatization was used to assess the relative importance of developmental goals within each country. The archival values demonstrated significant and positive correlations with corresponding self-reported values, validating their measurement. Supplementary analyses (Tables SS2 to SS3) confirmed the interrelationships among the values and their alignment with Schwartz's circular structure. To simplify conflicting dimensions, CON-OTC and SE-ST archive-based value-continuums were computed. Pearson correlations indicated significant associations between archive-based and self-reported value-continuums (CON-OTC: *r* = 0.57, *p* < 0.001; SE-ST: *r* = 0.49, *p* < 0.001). Fig. 7 illustrates the global distribution of archive-based value-continuums.

**Table 1:** The relationships between the archive-based and self-reported basic values in Study 2 (*N* = 89).

Archive-based basic values	Self-reported basic values										
	SE	CO	TR	SD	ST	HE	PO	AC	BE	UN	Mean correlation with noncorresponding self-reported values^a^
SE: Active military participation (*df* = 87)	.28**	.41***	.29**	−.41***	−.25**	−.36***	.25**	.40***	−.23*	−.39**	−.03
CO: Fertility rate and reversed age at first marriage (*df* = 89)	.07	.52***	.38***	−.38***	−.20*	−.41***	.30**	.61***	−.40***	−.49***	−.06
TR: Reversed female male labor participation ratio (*df* = 88)	.26**	.27**	.47***	−.46***	−.41***	.08	−.03	.41***	−.38***	−.34**	−.07
SD: H-index (*df* = 88)	−.09	−.33**	−.36***	.39***	.19*	.11	−.14^ɸ^	−.35***	.36***	.32**	−.00
ST: Movie production (*df* = 87)	−.13	−.32**	−.30**	.25**	.22*	.16	−.13	−.38***	.41***	.25**	−.02
HE: Daily intake of calories (*df* = 86)	−.02	−.41***	−.28**	.28**	.11	.30**	−.22*	−.50***	.34**	.41***	−.04
PO: United Nations peacekeepers (*df* = 73)	−.17^ɸ^	.29**	.16^ɸ^	−.13	.16^ɸ^	−.37**	.34**	.30**	−.30**	−.32**	−.04
AC: Gross domestic product growth rate (*df* = 89)	.08	.35***	.35***	−.42***	−.19*	−.04	.08	.49***	−.43***	−.28**	−.06
BE: Healthcare expenditure (*df* = 88)	−.03	−.27**	−.43***	.44***	.12	.27**	−.28**	−.42***	.37***	.35***	−.03
UN: EPI and reversed GINI index (*df* = 87)	−.10	−.42***	−.24*	.31**	.18*	.20*	−.11	−.55***	.27**	.43***	−.06

*Note*. SE = Security, CO = Conformity, TR = Tradition, SD = Self-direction, ST = Stimulation, HE = Hedonism, PO = Power, AC = Achievement, BE = Benevolence, UN = Universalism. GINI index measures income inequality and EPI is Environmental Performance Index. A = The mean correlation was computed using the Fisher's *Z* transformation and back transformation as described in Bardi et al. (2008). ^ɸ^*p* < 0.1, *p < 0.05, **p < 0.01, ***p < 0.001.

Importantly, no single behavioral indicator can fully capture values due to external influences, as Bardi and Schwartz (2003) note. The selected archival indicators only partially reflect corresponding motivational goals. However, compatibility between values emerged within the circular structure; for instance, active military participation indicated security and conformity values. Additionally, certain values shared general motivational goals like self-protection for conservation and self-enhancement. The archival conformity indicator correlated positively with self-reported achievement, while the achievement indicator correlated with self-reported conformity. Likewise, the anxiety-free growth values of self-direction, stimulation, benevolence, and universalism showed positive correlations between their archival and self-reported counterparts. These findings partially supported Schwartz et al.’s (2012) refined theory, suggesting the indicators capture some motivational value goals at the country level.

##### 3.1.2.2 Other variables

COVID-19 severity indicators, latitude, historical pathogen prevalence index, stringency index, and modernization index were the same as examined in Study 1.

### 3.2 Statistical approach

All statistical analyses were carried out as described in Study 1.

### 3.3 Results and discussion

**Fig. 8:**
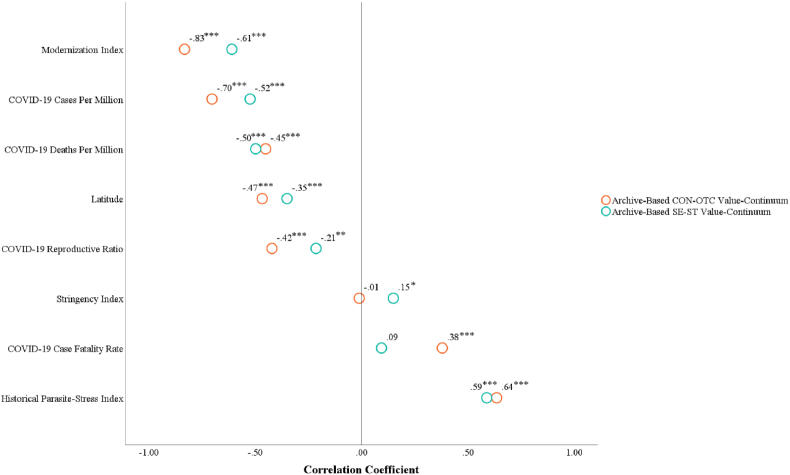
The correlations between country-level values and all variables in Study 2. Note. *p_one-tailed_ < 0.05, **p_one-tailed_ < 0.01, ***p_one-tailed_ < 0.001.

**Fig. 9:**
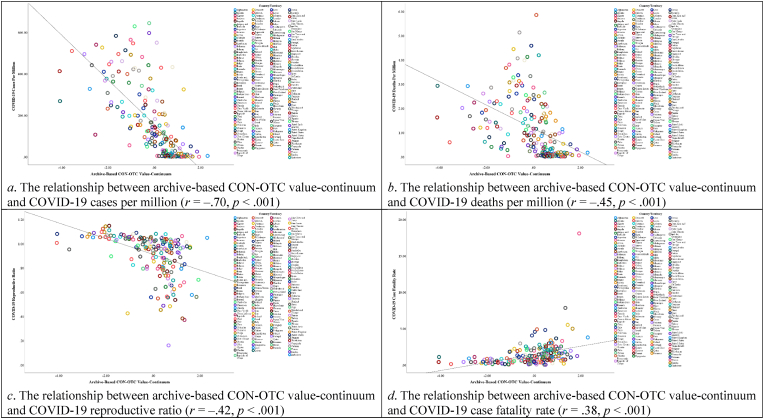
Scatter plots displaying the relationships between archive-based CON-OTC value-continuum and COVID-19 severity indicators in Study 2.

**Fig. 10:**
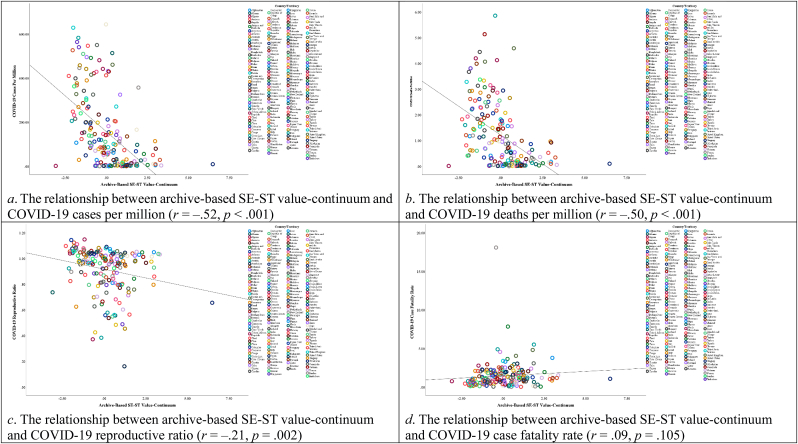
Scatter plots displaying the relationships between archive-based SE-ST value-continuum and COVID-19 severity indicators in Study 2.

Fig. 8 depicts the correlations between values and all variables, with detailed descriptive statistics and correlations in the Supplementary File's Table S3. As shown in Fig. 9, Fig. 10, the archive-based CON-OTC value-continuum exhibited significant negative associations with COVID-19 cases per million, deaths per million, and reproductive ratio, while positively correlating with case fatality rate. Similarly, the archive-based SE-ST value-continuum demonstrated significant negative relationships with cases per million, deaths per million, and reproductive ratio.

Next, multilevel analysis was conducted to examine the effects of archive-based values on COVID-19 severity indicators while controlling for all covariates. The unconditional multilevel models, with geopolitical regions as a random intercept, demonstrated that geopolitical regions accounted for a substantial amount of the variance in country-level COVID-19 cases per million (ICC = 49.9%, LRT = 86.9, *p* < .001), COVID-19 deaths per million (ICC = 51.4%, LRT = 123, *p* < .001), COVID-19 reproductive ratio (ICC = 54.0%, LRT = 69.6, *p* < .001), and COVID-19 case fatality rate (ICC = 8.79%, LRT = 4.15, *p* = .042). These results indicated the significance of multilevel analysis in understanding the impact of values on COVID-19 severity.

Regarding the association between archive-based values and COVID-19 cases per million (see Table S4), the results from the multilevel models showed that archive-based CON-OTC value-continuum had a significant negative prediction (B = −81.25, *SE* = 10.71, *df* = 177, *t* = −7.59, *p*_one-tailed_ < 0.001), as did archive-based SE-ST value-continuum (B = −22.23, *SE* = 7.65, *df* = 177, *t* = −2.90, *p*_one-tailed_ = 0.002). However, after controlling for covariates, archive-based CON-OTC value-continuum did not significantly predict COVID-19 cases per million (B = −8.24, *SE* = 14.06, *df* = 157, *t* = −0.59, *p*_one-tailed_ = 0.279), nor did archive-based SE-ST value-continuum (B = −6.28, *SE* = 8.60, *df* = 157, *t* = −0.73, *p*_one-tailed_ = 0.233).

In relation to COVID-19 deaths per million, the multilevel models indicated that archive-based CON-OTC value-continuum had a significant negative prediction before covariate control (B = −0.24, *SE* = 0.09, *df* = 177, *t* = −2.63, *p*_one-tailed_ = 0.004), but this effect became non-significant after accounting for covariates (B = −0.07, *SE* = 0.13, *df* = 157, *t* = −0.55, *p*_one-tailed_ = 0.292). On the other hand, archive-based SE-ST value-continuum was a significant predictor before controlling for covariates (B = −0.14, *SE* = 0.06, *df* = 177, *t* = −2.33, *p*_one-tailed_ = 0.020) and after controlling for covariates (B = −0.15, *SE* = 0.07, *df* = 157, *t* = −2.07, *p*_one-tailed_ = 0.020).

Furthermore, the multilevel models revealed that archive-based CON-OTC value-continuum significantly and negatively predicted COVID-19 reproductive ratio (B = −0.05, *SE* = 0.01, *df* = 176, *t* = −3.60, *p*_one-tailed_ < 0.001), but this effect did not hold true after accounting for covariates (B = −0.01, *SE* = 0.02, *df* = 156, *t* = −0.31, *p*_one-tailed_ = 0.378). Archive-based SE-ST value-continuum did not significantly predict COVID-19 reproductive ratio before covariate control (B = 0.01, *SE* = 0.01, *df* = 176, *t* = 1.26, *p*_one-tailed_ = 0.105), and it remained a non-significant predictor after covariate control (B = 0.01, *SE* = 0.01, *df* = 156, *t* = 1.14, *p*_one-tailed_ = 0.128).

Regarding COVID-19 case fatality rate, the multilevel models indicated that archive-based CON-OTC value-continuum significantly predicted this indicator (B = 0.70, *SE* = 0.12, *df* = 177, *t* = 5.58, *p*_one-tailed_ < 0.001), and this effect remained significant after controlling for covariates (B = 0.51, *SE* = 0.21, *df* = 157, *t* = 2.42, *p*_one-tailed_ = 0.008). Archive-based SE-ST value-continuum also significantly predicted COVID-19 case fatality rate (B = −0.22, *SE* = 0.10, *df* = 177, *t* = −2.11, *p*_one-tailed_ = 0.018), and this effect held true when controlling for covariates (B = −0.33, *SE* = 0.13, *df* = 157, *t* = −2.46, *p*_one-tailed_ = 0.007).

**Fig. 11:**
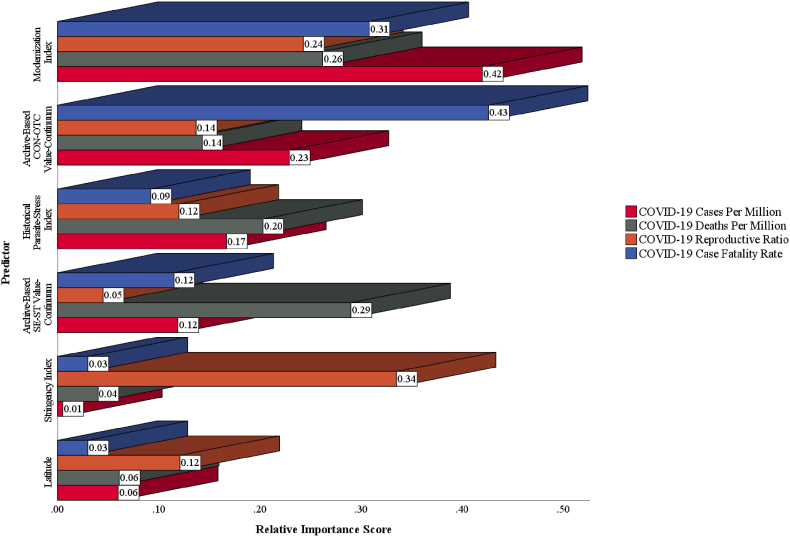
Relative importance of predictors for COVID-19 severity indicators in Study 2.

In support of the conclusion drawn in Study 1, Fig. 11 displays the relative importance scores, highlighting that Ma's modernization index (2020) contributed significantly to the understanding of the overall severity of COVID-19 pandemic. Additionally, the relative importance scores demonstrated that archive-based CON-OTC value-continuum had the highest importance as a predictor of COVID-19 case fatality rate (relative importance score = 0.43), while archive-based SE-ST value-continuum had the highest importance as a predictor of COVID-19 deaths per million (relative importance score = 0.29).

Based on the findings from Study 2, it appears that countries valuing conservation faced higher COVID-19 lethality, while openness-to-change-valuing countries saw lower severity. Moreover, after adjusting for the effects of covariates, self-enhancement values predicted relatively lower lethality and mortality burden, whereas self-transcendence values were linked to higher severity.

## 4 General discussion

This research provides evidence from two studies that country-level Schwartz's values partially predicted COVID-19 severity beyond previously identified predictors (Frey et al., 2020; Gangemi et al., 2020; Jankowiak et al., 2020; Notari, 2021; Roy & Ghosh, 2020; Sorci et al., 2020; Wang, 2021). Specifically, Study 1 showed conservation values slowed initial COVID-19 spread and were most influential for the reproductive ratio. However, both studies consistently revealed higher conservation countries suffered more severe COVID-19 impact, with higher case fatality rates. In contrast, findings from both studies consistently indicated openness-to-change countries experienced lower coronavirus severity and lethality, with reduced mortality risk among confirmed cases.

The findings for the CON-OTC value-continuum have two potential explanations. First, countries higher on conservation values prioritize tradition and conformity (Schwartz, 1994, 2012), which may have delayed adapting behaviors to manage COVID-19 and impeded public health innovations, increasing case fatality rates. Second, countries higher on openness to change values emphasize flexibility, novelty and change (Schwartz, 1994, 2012). Their adaptability likely enabled effective response, lowering severity. Specifically, their preparedness to innovate facilitated evidence-based interventions to reduce virus transmission and fatalities. Additionally, prioritizing science inclined them toward data-driven policies when managing the crisis.

Previous research hypothesized that prosocial values could mitigate COVID-19's impacts by promoting compliance and concern for others (Ma, 2022a; Wolf et al., 2020). However, the current study proposed that countries prioritizing greater self-interest and competitiveness would demonstrate lower COVID-19 severity, since these values serve self-protection motives (Schwartz, 2012; Schwartz et al., 2012). The findings across both studies support this hypothesis. Compared to nations emphasizing self-transcendence values, countries prioritizing self-enhancement values experienced relatively lower COVID-19 severity, as measured by prevalence rate, mortality rate, and reproductive ratio. Importantly, after accounting for modernization, latitude, historical pathogen prevalence, and policy strictness, results reveal that self-enhancement-oriented countries had relatively lower case fatality rates than those valuing self-transcendence. This aligns with self-enhancement values driving self-protection (Schwartz, 2012; Schwartz et al., 2012). Possible explanations include increased compliance with authorities, conformity to social norms, isolation willingness, and connections to vertical collectivism and personal COVID-19 concern in these cultures.

Firstly, self-enhancement values are associated with support for social hierarchy (Hofstede & McCrae, 2004; Leone et al., 2016). Thus, countries that prioritize self-enhancement likely exhibit higher power distance and acceptance of status differentiation (Schwartz, 2012; Schwartz et al., 2012). This implies greater compliance with authorities, including adherence to COVID-19 prevention guidelines and restrictions (Gokmen et al., 2021). In cultures emphasizing achievement and power, individuals may be more willing to follow top-down rules like lockdowns and mask mandates from respected leadership, resulting in mitigated viral transmission. The hierarchical nature of self-enhancement-oriented societies could promote conformity to COVID-19 containment measures, helping to reduce disease severity.

Secondly, self-enhancement values prioritize social esteem (Schwartz, 2012). In countries oriented this way, meeting standards like adhering to COVID-19 measures could affirm competence and capability (Schwartz, 2012). Thus, individuals may closely monitor their pandemic behaviors because conformity enhances status and esteem. This motivation to demonstrate competence and gain approval could encourage preventative actions that mitigate viral spread. In cultures emphasizing achievement and success, following COVID-19 guidelines may be viewed as an opportunity to gain admiration by showcasing responsibility and competence. Therefore, the desire for social esteem in self-enhancement-oriented societies could indirectly promote norm-abiding behaviors that reduce viral transmission.

Thirdly, the relationship between self-enhancement values and vertical collectivism, both of which prioritize status differentiation (Clay et al., 2012; Schwartz, 2012; Schwartz et al., 2012; Triandis, 1996), may also contribute to lower COVID-19 severity. Recent individual-level studies have demonstrated that higher levels of vertical collectivism are associated with increased personal concern about COVID-19 as well as greater intentions to practice social distancing (Biddlestone et al., 2020; Germani et al., 2020). Specifically, individuals in these self-enhancement-oriented societies may be more motivated to follow COVID-19 restrictions in order to maintain status and uphold group norms, partly explaining lower severity at the national level.

Fourthly, Tabernero et al. (2020) demonstrated that the basic value of power was associated with greater willingness for social distancing and more effective COVID-19 coping at the individual level. This implies that citizens in countries that emphasize power values could be more motivated to engage in preventive behaviors like distancing and hygiene adherence, with social esteem and competence validation driving virus severity mitigation at the national level. In self-enhancement-oriented cultures, individuals may view following COVID-19 restrictions as an opportunity to gain status and respect by demonstrating their competence.

Interestingly, there was a small positive correlation between self-enhancement values and the COVID-19 stringency index across studies. This suggests countries with higher self-enhancement values may be somewhat more inclined to adopt strict control measures, as reflected in the COVID-19 stringency index which measures government restrictions (Frey et al., 2020; Kapoor et al., 2021; Sorci et al., 2020; Wang, 2021). By implementing more stringent measures, countries emphasizing self-enhancement values could mitigate pandemic severity. However, compared to other predictors like modernization and values, the stringency index had a lower impact on COVID-19 outcomes across studies. Therefore, while self-enhancement values may relate to stricter control measures, the data indicate this plays a minor role in reducing COVID-19 severity relative to other factors. Future studies could unpack the nuanced relationship between societal values, government restrictions, and pandemic outcomes.

The findings linking country's value priorities and COVID-19 severity have salient implications for public health policy and messaging. They suggest campaigns could consider prevailing values when promoting behaviors in a given country. For instance, affirming traditional norms while still encouraging adaptive practices may resonate more in conservation-valuing nations. Additionally, policymakers could factor values into planning. For example, as openness-to-change countries are more receptive to innovation, policies advancing scientific literacy, research investment, and knowledge exchange could further capitalize on this adaptability. Furthermore, self-enhancement focused countries may be more responsive to messaging that emphasizes personal safety, empowerment, and self-protection. Overall, aligning interventions with country's value priorities can make them more effective during global health crises.

The present research provides valuable insights into Schwartz's personal values at a country level during the COVID-19 pandemic. It demonstrates strengths in utilizing large-scale data from reputable sources, incorporating multiple severity indicators, employing multilevel analysis, and innovatively using archival indicators. These strengths enhance the credibility, reliability, and generalizability of the findings while providing a broader societal perspective on values and enriching the research.

However, limitations exist. First, individual demographic data was excluded due to the country-level focus, and the modernization index may not fully capture nuanced demographic impacts. Accessing individual-level data could enable examination of age, gender, and other factors. Second, the observational data limits establishing causal links between values and COVID-19 severity, as unmeasured variables may influence the associations. Experimental or longitudinal designs could better assess causality. Finally, the limited archival indicators per value reduce comprehensive assessment of country-level values. Incorporating more indicators and replicating across cultures would enhance understanding of their predictive power.

## 5 Conclusion

Overall, this research serves as a strong foundation for future investigations by demonstrating the predictive utility of assessing Schwartz's values dimensions at the societal level. The findings offer practical implications for understanding how a country's predominant values orientation may influence COVID-19 outcomes. This interplay between country's value priorities and pandemic impact warrants deeper exploration and presents opportunities to inform public health strategies tailored to national values.

## Ethical statement

All data analyzed in the current research were obtained from publicly available datasets. A secondary analysis of publicly available data does not require Institutional Review Board approval according to the corresponding author's institution.

## CRediT authorship contribution statement

**Mac Zewei Ma:** Conceptualization, Methodology, Investigation, Formal analysis, Writing – original draft. **Shengquan Ye:** Writing – review & editing.

## Declaration of competing interest

The author(s) declared no potential conflicts of interest with respect to the research, authorship, and/or publication of this article.

## Data Availability

Research data can be found via this Open Science Framework (OSF) repository: https://osf.io/jgdfm/?view_only=c65131f8890c4419ba56a788e99f1084
