# Supplementary material for: Country's value priorities in health crisis: How dominant societal motivations shape COVID-19 severity
Source: SSM Popul Health. 2023 Aug 19;24:101493. doi: 10.1016/j.ssmph.2023.101493 (PMC10474233; doi:10.1016/j.ssmph.2023.101493)
Supplement: Multimedia component 1 [file mmc1.pdf]

Table S1. Bivariate correlation results in Study 1

Variable	n	M	SD	1. Self-reported CON-OTC Value-continuum	2. Self-reported SE-ST Value-continuum	3. Latitude	4. Historical parasite-stress	5. Modernization index	6. Stringency index	7. COVID-19 cases per million	8. COVID-19 deaths per million	9. Reproductive ratio
1. Self-reported CON-OTC Value-continuum	89	0.64	0.44									
2. Self-reported SE-ST Value-continuum	89	-0.98	0.53	.24*								
3. Latitude	89	28.8	25.72	-.20*	-.09							
4. Historical parasite-stress	89	-0.08	0.66	.47***	.45***	-.53***						
5. Modernization index	89	0.21	0.68	-.59***	-.56***	.44***	-.79***					
6. Stringency index	89	44.4	8.82	.25*	.15	-.25**	.38***	-.29**				
7. COVID-19 cases per million	89	211.79	180.37	-.56***	-.42***	.35***	-.59***	.81***	-.28**			
8. COVID-19 deaths per million	89	1.61	1.32	-.05	-.32***	.21*	-.39***	.39***	-.09	.38***		
9. Reproductive ratio	89	1.00	0.09	-.51***	-.25*	.15	-.28**	.50***	.24*	.37***	.29**	
10. Case fatality rate	89	1.43	2.05	.38***	.06	-.17	.21*	-.40***	-.13	-.39***	.02	-.39***

Note. $^{*}p < .05$, $^{**}p < .01$, $^{***}p < .001$.

Table S2. Multilevel analysis results in Study 1

Predictor	Outcome variable																															
	COVID–19 cases per million								COVID–19 deaths per million								COVID-19 reproductive ratio								COVID-19 case fatality rate							
	Model 1 without covariates				Model 2 with covariates				Model 1 without covariates				Model 2 with covariates				Model 1 without covariates				Model 2 with covariates				Model 1 without covariates				Model 2 with covariates			
	B	*SE*	*df*	*t*	B	*SE*	*df*	*t*	B	*SE*	*df*	*t*	B	*SE*	*df*	*t*	B	*SE*	*df*	*t*	B	*SE*	*df*	*t*	B	*SE*	*df*	*t*	B	*SE*	*df*	*t*
Constant	218.07	43.34	86	5.03***	268.74	69.37	81	3.87	0.90	0.38	86	2.39**	0.10	0.72	81	0.13	1.04	0.02	86	42.53***	0.82	0.05	81	18.16***	0.11	0.58	86	0.19	3.12	1.16	81	2.68**
Self-reported CON-OTC value-continuum	-173.64	36.68	86	-4.73***	-44.36	33.31	81	-1.33	0.23	0.31	86	0.76	0.67	0.34	81	1.97*	-0.10	0.02	86	-5.16***	-0.07	0.02	81	-3.35***	1.84	0.47	86	3.89***	1.18	0.55	81	2.17*
Self-reported SE-ST value-continuum	-86.00	29.60	86	-2.91***	7.78	25.83	81	0.30	-0.35	0.25	86	-1.42	0.03	0.26	81	0.10	-0.02	0.02	86	-1.43	0.002	0.02	81	0.13	-0.13	0.39	86	-0.34	-0.77	0.44	81	-1.77*
Latitude					0.09	0.57	81	0.15					0.00	0.01	81	0.61					0.0002	0.00	81	0.65					0.00	0.01	81	-0.47
Historical parasite-stress index					31.73	30.02	81	1.06					-0.18	0.32	81	-0.55					0.03	0.02	81	1.69*					-0.55	0.49	81	-1.13
Modernization index					217.12	31.38	81	6.92***					0.78	0.32	81	2.43**					0.08	0.02	81	3.84***					-1.67	0.53	81.00	-3.17**
Stringency index					-1.63	1.34	81	-1.22					0.01	0.01	81	1.08					0.005	0.00	81	5.34***					-0.06	0.02	81.00	-2.75**

Note. $^{*}p < .05$, $^{**}p < .01$, $^{***}p < .001$.

Table S3. Bivariate correlation results in Study 2

Variable	*n*	*M*	*SD*	1. Archive-based CON-OTC Value-continuum	2. Archive-based SE-ST Value-continuum	3. Latitude	4. Historical parasite-stress	5. Modernization index	6. Stringency index	7. COVID-19 cases per million	8. COVID-19 deaths per million	9. Reproductive ratio
1. Archive-based CON-OTC Value-continuum	190	-0.04	1.13									
2. Archive-based SE-ST Value-continuum	180	0.09	1.38	.58***								
3. Latitude	227	18.13	24.51	-.47***	-.35***							
4. Historical parasite-stress	211	0.01	0.64	.64***	.59***	-.33***						
5. Modernization index	205	0.00	0.80	-.83***	-.61***	.46***	-.76***					
6. Stringency index	179	41.80	11.44	-.01	.15*	-.06	.10	-.03				
7. COVID-19 cases per million	227	181.05	177.73	-.70***	-.52***	.35***	-.61***	.76***	-.10			
8. COVID-19 deaths per million	227	1.14	1.17	-.45***	-.50***	.35***	-.38***	.50***	.07	.45***		
9. Reproductive ratio	190	0.89	0.20	-.42***	-.21**	.38***	-.22**	.44***	.36***	.28***	.40***	
10. Case fatality rate	227	1.27	1.58	.38***	.09	-.06	.30***	-.35***	-.04	-.41***	.03	-.06

Note. $^{*}p < .05$, $^{**}p < .01$, $^{***}p < .001$.

Table S4. Multilevel analysis results in Study 2

Predictor	Outcome variable																															
	COVID–19 cases per million								COVID–19 deaths per million								COVID-19 reproductive ratio								COVID-19 case fatality rate							
	Model 1 without covariates				Model 2 with covariates				Model 1 without covariates				Model 2 with covariates				Model 1 without covariates				Model 2 with covariates				Model 1 without covariates				Model 2 with covariates			
	B	*SE*	*df*	*t*	B	*SE*	*df*	*t*	B	*SE*	*df*	*t*	B	*SE*	*df*	*t*	B	*SE*	*df*	*t*	B	*SE*	*df*	*t*	B	*SE*	*df*	*t*	B	*SE*	*df*	*t*
Constant	144.58	14.92	177	9.69***	210.19	37.28	157	5.64***	1.09	0.17	177	6.43***	0.44	0.35	157	1.25	0.92	0.02	176	43.43***	0.67	0.05	156	12.78***	1.49	0.11	177	13.01***	1.75	0.58	157	3.01**
Self-reported CON-OTC value-continuum	-81.25	10.71	177	-7.59***	-8.24	14.06	157	-0.59	-0.24	0.09	177	-2.63**	-0.07	0.13	157	-0.55	-0.05	0.01	176	-3.60***	-0.01	0.02	156	-0.31	0.70	0.12	177	5.58***	0.51	0.21	157	2.42**
Self-reported SE-ST value-continuum	-22.23	7.65	177	-2.90**	-6.28	8.60	157	-0.73	-0.14	0.06	177	-2.33*	-0.15	0.07	157	-2.07*	0.01	0.01	176	1.26	0.01	0.01	156	1.14	-0.22	0.10	177	-2.11*	-0.33	0.13	157	-2.46**
Latitude					-0.13	0.44	157	-0.30					0.00	0.00	157	0.63					0.00	0.00	156	2.12*					0.00	0.01	157	0.47
Historical parasite-stress index					-3.73	22.17	157	-0.17					-0.19	0.20	157	-0.92					-0.01	0.03	156	-0.31					-0.07	0.34	157	-0.20
Modernization index					176.43	27.93	157	6.32***					0.27	0.26	157	1.06					0.08	0.04	156	2.02*					-0.68	0.42	157	-1.64
Stringency index					-1.01	0.79	157	-1.27					0.01	0.01	157	2.04*					0.01	0.00	156	4.78***					-0.01	0.01	157	-0.60

Note. $^{*}p < .05$, $^{**}p < .01$, $^{***}p < .001$.

Selection of the 10 Archival Indicators Measuring Country-level Values

To overcome the limitations of self-reported data (Bardi et al., 2008), this study utilized archival indicators to measure group-level values. Archival indicators were selected based on the theoretical definitions of each basic value (Schwartz, 1994). Table SS1 shows the archival indicator of each basic value.

Table SS1. Archival indicator of each basic value in Study 2

Basic values	Motivational goals	Archival indicator	Data source	Rationale behind including the specific archival indicator(s)
Security	Safety, harmony, and stability of society, of relationships, and of self	Active military per 1,000 capita	https://www.iiss.org/	Military personnel per capita was used to represent security values, which emphasize safety, stability, and order (Schwartz, 1994). Military forces maintain national security, aligning with security motivations.
Conformity	Restrain impulses and inhibit actions to avoid violating social norms and expectations	Average age at first marriage (reversed); Fertility rate	https://data.worldbank.org/ https://en.wikipedia.org/wiki/List_of_countries_by_age_at_first_marriage	Conformity values inhibit impulses to avoid norm violations, enabling coalition building and reproductive fitness per evolutionary theory (Schwartz & Rubel-Lifschitz, 2009). Fertility and earlier marriage relate to fitness (Asadullah & Wahhaj, 2019; Munshi & Myaux, 2006; Perelli-Harris, 2005). Thus, fertility rates and age at first marriage represent conformity values, with higher fertility and younger marriage ages indicating conformity emphasis.
Tradition	Respect, commitment, and acceptance of the customs and ideas that traditional culture or religion provide	Female to male labor force participation rate (%)	https://data.worldbank.org/	Traditional cultures and religious beliefs affect female workforce participation, as they often limit (Contreras & Plaza, 2010; Dildar, 2015; H'madoun, 2010). Thus, lower female to male workforce ratios represent tradition values through women's workforce disengagement.
Self-direction	Independent thought and action, which are manifested through choosing, creating and exploring	H-index	https://www.scimagojr.com/	Academic research enables self-direction through creativity and curiosity (Bardi et al., 2008; Schwartz, 2012). Thus, the H-index, based on publication and citation rates (Hirsch, 2005), represents national self-direction values through research productivity and impact (Jacsó, 2009).

Stimulation	Excitement, novelty, and challenge in life	Films released per capita	https://www.the-numbers.com/movies/production-countries/#tab=territory	Watching entertainment films fulfill the goals of stimulation (Bardi et al., 2008)
Hedonism	Pleasure and sensuous gratification for oneself	Daily caloric supply per capita;<br>Consumption of fish and meat per capita	http://www.fao.org/faostat/en/#data/FBS<br>York and Gossard (2004)	Eating fulfills hedonism values by activating brain reward systems, satisfying sensuous needs (Kringelbach, 2015). For instance, taste strongly drives potato chip purchases for people prioritizing hedonism (Kitsawad & Guinard, 2014).
Power	Social status and prestige, control or dominance over people and resources	United Nation Peacekeeping force	https://peacekeeping.un.org/en/troop-and-police-contributors	Major conflicts challenge state authority (Gorur & Vellturo, 2017) by destabilizing politics and creating tensions. UN peacekeeping troops help manage local conflicts (Gorur & Vellturo, 2017) and restore state authority (Jones, 2009; Murphy, 2016). Thus, UN peacekeeper contributions represent power values emphasizing authority.
Achievement	Strive for success through demonstrating competence according to social standards	GDP growth rate	https://data.worldbank.org/	GDP growth rate indicates economic expansion, aligning with demonstrating successful performance per achievement definitions (Schwartz, 2012). Growth results from year-over-year comparison, consistent with achievement value processes (Schwartz et al., 2012). Greater growth often sacrifices environmental sustainability, reflecting achievement's self-centered motivations (Marjanović et al., 2016; Schwartz, 2012).
Benevolence	Preserve and enhance the welfare of people with whom one is in frequent personal contact	Healthcare expenditure (% of GDP)	https://data.worldbank.org/	While no indicator perfectly measures helping ingroup members, healthcare spending represents benevolence values by promoting citizen health (Rahman et al., 2018), aligning with the definition of benevolence (Schwartz, 2012). For instance, higher spending correlates with improved health outcomes like lower infant mortality, advancing wellbeing (Patton et al., 2016; Rahman et al., 2018)
Universalism	Understand, appreciate, tolerate, and protect	GINI Index (reversed);	https://www.cia.gov/index.html	The GINI index (reversed) represents universalism by measuring income inequality, with lower GINI indicating greater equality (Bosi & Seegmuller, 2006). The Environmental Performance Index also represents universalism through its sustainability and nature protection metrics (Hsu & Zomer, 2014).

	for the welfare of all people and for nature	Environmental<br><br>Performance Index	https://epi.yale.edu/	

Additional Tests for the Validity of the Archival Measure

The interrelationships among the ten basic values showed highly similar patterns across the self-report and archival data, although the archival correlations were larger (see Table SS2). For instance, conformity negatively correlated with self-transcendence values like benevolence and universalism in both data sets, but archival measures revealed stronger associations ($-.58 \leq r \leq -.52$ vs. $-.19 \leq r \leq -.18$). Analyses of the mean correlations between values within the same higher-order dimensions found no significant differences between self-reported and archival data. Specifically, the average correlations between conservation values were $r = .28$ and $r = .42$; between openness values $r = .18$ and $r = .28$; between self-enhancement values $r = .31$ and $r = .35$; and between self-transcendence values $r = .55$ and $r = .37$. While archival measures showed somewhat stronger relationships, these differences were statistically non-significant. Overall, the archival indicators seem to have adequately captured the essential interrelationships between the ten basic values, providing validity evidence. The similar patterns across data sources suggest the archived indicators can ecologically assess societal values and their motivational structures.

Table SS2. The interrelationships among the basic values

Values	1. SE	2. CO	3. TR	4. SD	5. ST	6. HE	7. PO	8. AC	9. BE	10. UN
1. SE		$.17_{n = 90}$	$.13_{n = 90}$	$-.25^{*}_{n = 90}$	$-.54^{***}_{n = 90}$	$-.24^{*}_{n = 90}$	$-.11_{n = 90}$	$.20^{\phi}_{n = 90}$	$.01_{n = 90}$	$-.07_{n = 90}$
2. CO	$.65^{***}_{n = 88}$		$.51^{***}_{n = 90}$	$-.49^{***}_{n = 90}$	$-.27^{**}_{n = 90}$	$-.48^{***}_{n = 90}$	$-.12_{n = 90}$	$.42^{***}_{n = 90}$	$-.18^{\phi}_{n = 90}$	$-.19^{\phi}_{n = 90}$
3. TR	$.20^{\phi}_{n = 88}$	$.36^{**}_{n= 89}$		$-.49^{***}_{n = 90}$	$-.50^{***}_{n = 90}$	$-.22^{*}_{n = 90}$	$-.17_{n = 90}$	$.27^{*}_{n = 90}$	$-.25^{*}_{n = 90}$	$-.04_{n = 90}$
4. SD	$-.51^{***}_{n = 87}$	$-.56^{***}_{n = 89}$	$-.45^{***}_{n = 88}$		$.43^{***}_{n = 90}$	$.14_{n = 90}$	$-.15_{n = 90}$	$-.56^{***}_{n = 90}$	$.24^{*}_{n = 90}$	$.39^{***}_{n = 90}$
5. ST	$-.42^{***}_{n = 87}$	$-.44*^{**}_{n= 88}$	$-.43^{***}_{n = 88}$	$.02_{n = 87}$		$-.06_{n = 90}$	$.28^{**}_{n = 90}$	$-.26^{*}_{n = 90}$	$-.16_{n = 90}$	$-.02_{n = 90}$
6. HE	$-.37^{***}_{n = 86}$	$-.78^{***}_{n= 87}$	$-.42^{***}_{n = 87}$	$.46^{***}_{n = 86}$	$.35^{**}_{n = 86}$		$-.38^{***}_{n = 89}$	$-.32^{***}_{n = 90}$	$.01_{n = 90}$	$.13_{n = 90}$
7. PO	$.11_{n = 74}$	$.53^{***}_{n = 74}$	$.13_{n = 74}$	$-.40^{***}_{n = 74}$	$-.53^{***}_{n = 73}$	$-.68^{***}_{n = 74}$		$.31^{**}_{n = 90}$	$-.47^{***}_{n = 90}$	$-.65^{***}_{n = 90}$
8. AC	$.20^{\phi}_{n = 88}$	$.45^{***}_{n = 90}$	$.32^{*}_{n = 89}$	$-.47^{***}_{n = 89}$	$-.29^{**}_{n = 88}$	$-.47^{***}_{n = 87}$	$.41^{***}_{n = 74}$		$-.50^{***}_{n = 90}$	$-.64^{***}_{n = 90}$
9. BE	$-.23^{*}_{n = 88}$	$-.52^{***}_{n= 89}$	$-.46^{***}_{n= 88}$	$.40^{***}_{n = 88}$	$.22^{*}_{n = 87}$	$.48^{***}_{n = 86}$	$-.64^{***}_{n = 74}$	$-.58^{***}_{n = 89}$		$.56^{***}_{n = 90}$
10. UN	$-.14_{n = 87}$	$-.58^{***}_{n = 88}$	$-.34^{**}_{n= 88}$	$.22^{*}_{n = 87}$	$.17_{n = 87}$	$.52^{***}_{n = 87}$	$-.48^{***}_{n = 74}$	$-.58^{***}_{n = 88}$	$39^{***}_{n = 87}$	

Note. SE = Security, CO = Conformity, TR = Tradition, SD = Self-direction, ST = Stimulation, HE = Hedonism, PO = Power, AC = Achievement, BE = Benevolence, UN = Universalism. $^{\phi}p < .1$, $^{*}p < .05$, $^{**}p < .01$, $^{***}p < .001$

Examining correlations between the archival value indicators and corresponding self-report higher-order dimensions provided evidence for convergent validity (see Table SS3). The three conservation indicators related positively to self-reported conservation ($.45 \leq r \leq .48$, all *p*s < .001), with a higher mean correlation than with noncorresponding dimensions ($r = .46$ vs. $r = -.18$). The openness indicators also correlated positively with self-reported openness ($.29 \leq r \leq .36$, all *p*s < .01), again higher than with noncorresponding dimensions ($r = .32$ vs. $r = -.10$). Self-enhancement indicators positively correlated with self-reported self-enhancement ($.30 \leq r \leq .40$, all *p*s < .001), exceeding noncorresponding correlations ($r = .35$ vs. $r = -.13$). Finally, self-transcendence indicators related positively to self-reported self-transcendence ($.39 \leq r \leq .41$, all *p*s < .001), surpassing noncorresponding correlations ($r = .40$ vs. $r = -.13$). At the higher-order level, archival conservation correlated positively with self-reported conservation ($r = .60$, $p < .001$) and negatively with openness ($r = -.56$, $p < .001$). Archival openness associated positively with self-reported openness ($r = .44$, $p < .001$) and negatively with conservation ($r = -.51$, $p < .001$). Archival self-enhancement showed positive and negative relationships with self-reported self-enhancement ($r = .38$, $p < .001$) and self-transcendence ($r = -.41$, $p < .001$), respectively. Archival self-transcendence correlated positively and negatively with self-reported self-transcendence ($r = .47$, $p < .001$) and self-enhancement ($r = -.43$, $p < .001$), respectively. Overall, these findings demonstrate satisfactory convergent and discriminant validity for the archival value indicators and dimensions, supporting their ability to capture Schwartz's circular motivational structure at the country level.

Table SS3. The correlations between the archive-based basic values and their corresponding self-reported higher-order dimensions

Archive-based basic values	Self-report higher-order dimensions				
	CON	OTC	SE	ST	Mean correlation with noncorresponding higher-order dimensions[a]
SE: Active military participation (*df* = 87)	.45***	-.52***	.38***	-.36***	-.18
CO: Fertility rate and reversed age at first marriage (*df* = 89)	.48***	-.51***	.52***	-.50***	-.18
TR: Reversed female male labor participation ratio (*df* = 88)	.47***	-.33**	.18*	-.41***	-.19
SD: H-index (*df* = 88)	-.37***	.32**	-.28**	.36***	-.10
ST: Movie production (*df* = 87)	-.36***	.29**	-.28**	.36***	-.10
HE: Daily intake of calories (*df* = 86)	-.35***	.36***	-.40***	.43***	-.11
PO: United Nations peacekeepers (*df* = 73)	.15ᶲ	-.24*	.40***	-.33**	-.15
AC: Gross domestic product growth rate (*df* = 89)	.37***	-.29**	.30**	-.39***	-.10
BE: Healthcare expenditure (*df* = 88)	-.36***	.41***	-.41***	.41***	-.14
UN: Reversed GINI index and EPI (*df* = 87)	-.34**	.31**	-.33**	.39***	-.12

Note. SE = Security, CO = Conformity, TR = Tradition, SD = Self-direction, ST = Stimulation, HE = Hedonism, PO = Power, AC = Achievement, BE = Benevolence, UN = Universalism. a = The mean correlation was computed using the Fisher's *Z* transformation and back transformation as described in Bardi et al. (2008). Degrees of freedom

(*df*) refer to the correlations between the archive-based basic values and their corresponding self-report higher-order values dimensions. $^{\phi}p < .1$, $^{*}p < .05$, $^{**}p < .01$, $^{***}p < .001$.

References

Asadullah, M. N., & Wahhaj, Z. (2019). Early marriage, social networks and the transmission of norms. *Economica*, *86*(344), 801-831.

Bardi, A., Calogero, R. M., & Mullen, B. (2008). A new archival approach to the study of values and value--Behavior relations: Validation of the value lexicon. *Journal of Applied Psychology*, *93*(3), 483-497.

Bosi, S., & Seegmuller, T. (2006). Optimal cycles and social inequality: What do we learn from the Gini index? *Research in Economics*, *60*(1), 35-46.

Contreras, D., & Plaza, G. (2010). Cultural factors in women's labor force participation in Chile. *Feminist Economics*, *16*(2), 27-46.

Dildar, Y. (2015). Patriarchal norms, religion, and female labor supply: Evidence from Turkey. *World Development*, *76*, 40-61.

Gorur, A., & Vellturo, M. (2017). *Local conflict, local peacekeeping*. Stimson Center.

H'madoun, M. (2010). *Religion and labor force participation of women* [Working Paper 2010-007]. University of Antwerp, Faculty of Applied Economics. http://www.aiel.it/page/old_paper/Hmadoun.pdf

Hirsch, J. E. (2005). An index to quantify an individual's scientific research output.

Proceedings of the National Academy of Sciences, *102*(46), 16569-16572.

Hsu, A., & Zomer, A. (2014). Environmental performance index. *Wiley StatsRef: Statistics Reference Online*, 1-5.

Jacsó, P. (2009). The h-index for countries in Web of Science and Scopus. *Online Information Review*, *33*(4), 831-837.

Jones, B. (2009). Peacekeeping in Crisis? Confronting the Challenges Ahead. *The RUSI Journal*, *154*(5), 78-83.

Kitsawad, K., & Guinard, J.-X. (2014). Combining means-end chain analysis and the Portrait Value Questionnaire to research the influence of personal values on food choice. *Food Quality and Preference*, *35*, 48-58.

Kringelbach, M. L. (2015). The pleasure of food: underlying brain mechanisms of eating and other pleasures. *Flavour*, *4*(1), 1-12.

Marjanović, V., Milovančević, M., & Mladenović, I. (2016). Prediction of GDP growth rate based on carbon dioxide (CO2) emissions. *Journal of CO2 Utilization*, *16*, 212-217.

Munshi, K., & Myaux, J. (2006). Social norms and the fertility transition. *Journal of Development Economics*, *80*(1), 1-38.

Murphy, R. (2016). UN Peacekeeping in the Democratic Republic of the Congo and the

Protection of Civilians. *Journal of Conflict and Security Law*, *21*(2), 209-246.

Patton, G. C., Sawyer, S. M., Santelli, J. S., Ross, D. A., Afifi, R., Allen, N. B., Arora, M., Azzopardi, P., Baldwin, W., & Bonell, C. (2016). Our future: a Lancet commission on adolescent health and wellbeing. *The Lancet*, *387*(10036), 2423-2478.

Perelli-Harris, B. (2005). The path to lowest-low fertility in Ukraine. *Population Studies*, *59*(1), 55-70.

Rahman, M. M., Khanam, R., & Rahman, M. (2018). Health care expenditure and health outcome nexus: new evidence from the SAARC-ASEAN region. *Globalization and Health*, *14*(1), 113.

Schwartz, S. H. (1994). Are there universal aspects in the structure and contents of human values? *Journal of Social Issues*, *50*(4), 19-45.

Schwartz, S. H. (2012). An overview of the Schwartz theory of basic values. *Online Readings in Psychology and Culture*, *2*(1).

Schwartz, S. H., Cieciuch, J., Vecchione, M., Davidov, E., Fischer, R., Beierlein, C., Ramos, A., Verkasalo, M., Lönnqvist, J.-E., & Demirutku, K. (2012). Refining the theory of basic individual values. *Journal of Personality and Social Psychology*, *103*(4), 663-688.

Schwartz, S. H., & Rubel-Lifschitz, T. (2009). Cross-national variation in the size of sex

differences in values: Effects of gender equality. *Journal of Personality and Social Psychology*, *97*(1), 171-185.

York, R., & Gossard, M. H. (2004). Cross-national meat and fish consumption: exploring the effects of modernization and ecological context. *Ecological Economics*, *48*(3), 293-302.
